# Audiogram Comparison of Workers from Five Professional Categories

**DOI:** 10.1155/2015/201494

**Published:** 2015-02-03

**Authors:** Alexandre Scalli Mathias Duarte, Alexandre Caixeta Guimarães, Guilherme Machado de Carvalho, Laíza Araújo Mohana Pinheiro, Ronny Tah Yen Ng, Marcelo Hamilton Sampaio, Everardo Andrade da Costa, Reinaldo Jordão Gusmão

**Affiliations:** Occupational-Otolaryngological Medical Service, Department of Otolaryngology, Head and Neck Surgery, Rua Vital Brasil 251, School of Medical Sciences (FCM), University of Campinas (Unicamp), 13083-888 Campinas, SP, Brazil

## Abstract

*Introduction*. Noise is a major cause of health disorders in workers and has unique importance in the auditory analysis of people exposed to it. The purpose of this study is to evaluate the arithmetic mean of the auditory thresholds at frequencies of 3, 4, and 6 kHz of workers from five professional categories exposed to occupational noise. *Methods*. We propose a retrospective cross-sectional cohort study to analyze 2.140 audiograms from seven companies having five sectors of activity: one footwear company, one beverage company, two ceramics companies, two metallurgical companies, and two transport companies. *Results*. When we compared two categories, we noticed a significant difference only for cargo carriers in comparison to the remaining categories. In all activity sectors, the left ear presented the worst values, except for the footwear professionals (*P* > 0.05). We observed an association between the noise exposure time and the reduction of audiometric values for both ears. Significant differences existed for cargo carriers in relation to other groups. This evidence may be attributed to different forms of exposure. A slow and progressive deterioration appeared as the exposure time increased.

## 1. Introduction

Noise is considered the third major cause of environmental pollution and it may be seen as a risk factor of worsening health conditions. It becomes more complex when dealing with noise in the work environment due to its intensity, exposure time, and other risk factors [1]. When noise is intense and the exposure to it is continuous, structural changes may appear in the inner ear, which can lead to noise-induced hearing loss (NIHL). The exposure to physical, chemical, and organizational agents is considered a risk factor for work-related accidents and the noise is considered the most frequent aggressive physical agent in the work environment [2].

In USA, NIHL is the most common occupational disease. Approximately 30 million workers, in Europe and in the United States, are exposed to a potentially harmful noise level in their work environment [3]. In developing countries, the situation is usually more severe. Workers are commonly exposed to intense levels of noise and the use of hearing protection devices is often irregular [4].

It is consensus that the exposure time to noise is associated with audiological changes and NIHL. In the industrial district of Maracanaú, in the Brazilian State of Ceará, a study evaluated the audiometric profile of 5,372 workers of many industrial activities. The study identified 19% of occupational NIHL and showed that the hearing loss index differs in relation to noise exposure time [5].

In a study conducted with bus drivers in the city of Campinas, in the Brazilian State of São Paulo, a positive association between NIHL and noise exposure time was found [6]. Furthermore,epidemiological studies reveal that occupational hearing disorders affect more frequently professionals from metallurgical, mechanics, printing, textile, chemical/petrochemical, transport, and food and beverage companies [7]. It is known that the physical characteristics of noise (type, spectrum, and sound pressure level), the exposure time, and the individual susceptibility to noise can influence the risk of hearing disorders [8].

According to a study conducted in the city of Goiânia, in the Brazilian State of Goiás, the analysis of the hearing status of 187 metallurgists indicated the occurrence of hearing disorders: 21% suggesting occupational NIHL, 72% of normal conditions, and 7% suggesting other diseases. The study also analyzed the hearing status of 152 workers from a marble manufacturer company. These workers presented an average of 8.3 years of occupational exposure to noise. The results showed that 48% of the workers presented hearing loss, with a higher degree of loss at 6,000 Hz. Among the hearing alterations, 50% presented occupational NIHL whereas 41% were in the early stage of occupational NIHL [8].

According to a study conducted in the Federal District of Brazil, which investigated metallurgists, timber frame manufacturers, and marble manufacturers, it was observed that timber frame manufacturers are the workers that make the less use of hearing protection devices. Almost half of workers, 48.1%, reported that they do not use hearing protection devices, while 29.6% of them use it rarely. The index of workers with audiometric notch also varied according to the company: 53.8% of metallurgists, 48.1% of timber frame manufacturers, and 40.4% of marble manufacturers. According to environmental evaluation, there were observed differences between the noise spectrums in the environment. In the metallurgical company, the 8,000 Hz frequency band showed the most intense white noise level (85.5 dB HL), in the timber frame company, the prevailing frequency band was 2,000 Hz with noise level of 80.5 dB (HL), and in the marble manufacturer company, the prevailing frequency band was 4,000 Hz with white noise level of 79.3 dB (HL) [9].

Considering the importance of the problem, as well as the existence of methods of early detection and the lack of similar studies in the literature [10], our study aims to evaluate the arithmetic means of the hearing thresholds at frequencies of 3, 4, and 6 kHz of workers in various industrial sectors and relate them to the time of exposure to noise.

## 2. Materials and Methods

This is a cross-sectional study, in which retrospective data were collected in a specialized clinic in occupational medicine. Seven companies of the State of São Paulo were divided into five sectors of activity: one footwear company, one beverage company, two ceramics companies, two metallurgical companies, and two transport companies. All companies adopted programs on hearing preservation, according to Brazilian rules.

In the study, we included all audiometric examinations performed between January 2000 and January 2010 in the above-mentioned companies for all workers, totaling 18,973 exams. In the analysis, we used only the most recent audiometry from each worker. We did not use audiometries in which the auditory rest time was lower than 14 hours, as well as audiometries in which the arithmetic means of the hearing thresholds at frequencies of 500, 1,000, and 2,000 Hz were higher than 25 dB (HL) in any ear. Our purpose was to exclude any hearing impairments not related to noise exposure. We also excluded from the study workers with administrative duties or professionals who worked in places where they were not exposed to noise. After those procedures, there remained 2,140 audiograms for analysis.

**Figure 1:**
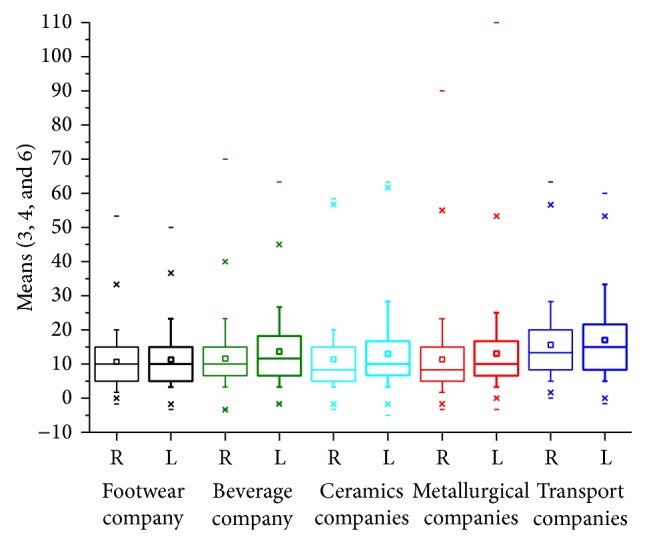
Box-plot of arithmetic means, in dB (HL), of the tonal thresholds at the frequencies of 3, 4, and 6 kHz for each ear and function.

We calculated the arithmetic means, in dB (HL), for the tonal thresholds at the audiometric frequencies of 3, 4, and 6 kHz for each ear (Figure 1). The selected workers were classified into four exposure groups: Group I, up to 60 months of exposure to noise; Group II, 61–120 months; Group III, 121–180 months, and Group IV, exposure of more than 180 months. We compared each ear among workers in terms of professional areas. Was compared hearing loss in the right and left ears and the association with age and duration of noise exposure (Tables 2 and 3).

For the statistical analysis, we used SAS System for Windows (version 9.2) (Table 1). The tests were bilateral and the significance level adopted was *P* < 0.05.

The study was approved by the Ethics and Research Committee of the University of Campinas (Report CEP/FCM no. 1161/2011).

## 3. Results

**Figure 2:**
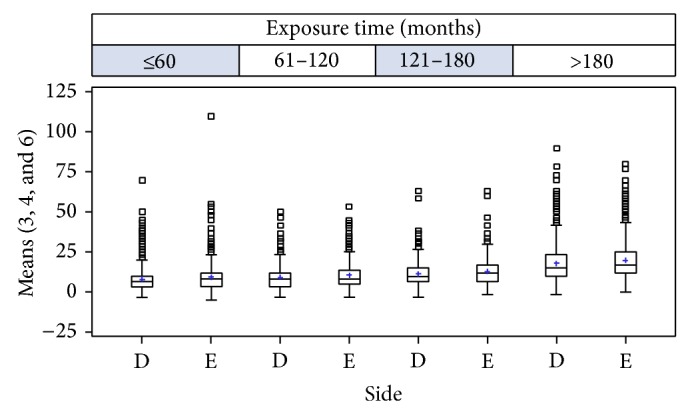
Box-plot of the arithmetic means, in dB (HL), of the tonal thresholds at the frequencies of 3, 4, and 6 kHz on each side and noise exposure range.

From the analysis of 2,140 audiometries, 1254 (58.60%) were from the metallurgical company, 266 (12.43%) from the footwear company, 236 (11.03%) from transport companies, 234 (10.93%) from the ceramics companies, and 150 (7.01%) from beverage company. The mean duration of noise exposure was 133.46 months (sd = 106.98; median = 111) and the mean age of the workers was 33.34 years (sd = 9.95; median = 32). The analysis of the means of the tonal thresholds at the frequencies of 3, 4, and 6 kHz in the right side was 11.79 dB (HL) (sd = 10.33 and median = 10) and in the left side it was 13.29 dB (HL) (sd = 10.85 and median = 10) (Figure 2).

Comparing the professional categories, the means of the tonal thresholds at the frequencies of 3, 4, and 6 kHz for each ear were higher in transport companies: 17.03 dB (HL) in the left ear. Comparing two isolated categories, a significant difference appeared only for the transport companies when they are compared to the others (*P* < 0.0001). For all professional areas, the left ear presented the worst values, with a significant *P*, except in the footwear company.

We observed a significant association between audiometric means and age: the higher the age, the higher the audiometric values. Additionally, the left side presented higher values than the right side at all times, for all ranges (*P* < 0.0001).

Some differences were observed between the audiograms of workers of different professional categories, and we also found worse hearing levels in the left ear for almost all categories. The worse hearing levels in left ear were shown only in few articles in medical literature and this is an important data about the asymmetry of occupational noise-induced hearing loss.

We observed an association between the noise exposure time and the audiometric values. We also observed that the left side presented higher values than the right side, in all ranges (*P* < 0.0001). In the comparison among categories, there was a progressive worsening of mean values, and statistical significance existed between Groups I and IV. We carried out a multiple analysis in order to investigate the factors that could have interfered in audiograms, as age and exposure time, and we verified that both the age range and the exposure time are associated with audiometric loss, together or separately.

## 4. Discussion

The World Health Organization (WHO) estimates that 10% of the world's population is exposed to high levels of sound pressure that can potentially lead to noise-induced hearing loss and is considered a public health problem. In the US, NIHL is the most common occupational disease [11].

The ongoing aggressive industrial development and the need for constant, fast, and efficient production raise special attention to the health of workers. Exposure to noise not only implies auditory changes, but also several extra-auditory effects.

Auditory reduction may interfere in the quality of life of workers, and it can lead to limitation in activities and restricted participation through the reduction in speech perception in noisy environments, television, radio, movie theaters, theaters, warning sound, music, and background music. Auditory reduction may lead to psychosocial consequences, such as stress and anxiety, and it can deteriorate social life in family, at work, and in the society in general [8–22].

Exposure to noise, occupational or not, is increasing more and more and it is linked to auditory symptoms (hearing loss, tinnitus, difficulty understanding speech, and hyperacusis) and nonauditory symptoms (irritation, sleep disorders, and cardiovascular diseases) [23–27].

In all the professional areas studied, except for footwear company, the hearing levels of the left ear were worse than those observed for the right ear. There are no clear technical reason for this difference between the sides, and we believed that the workers from transport companies could be more affected on the left ear considering that the noise in the left ear could be more intense than in the right ear because of the proximity of the window while driving trucks; however for the other professional areas there are no clear explanations for a different level of noise between ears.

The asymmetry of noise-induced hearing loss was already observed in previous studies [28–30]. The causes for this asymmetry can be attributed to the cortical pathways, specifically to the more pronounced efferent auditory system on the right side, which reduces the susceptibility of the right ear to cochlear insult, to the head shadow effect, and to physiological differences [29, 30].

## 5. Conclusion

In a comparative study of the audiometric analysis of workers from five different professional categories, the following were observed.

- Although mathematically incorrect, but universally adopted, the arithmetic means, of the tonal thresholds at the frequencies of 3, 4, and 6 kHz, in decibels, may be considered as a reference that indicates cochlear lesion due to a continuous exposure to intense noise.
- There were observed significant differences for the arithmetic means at 3, 4, and 6 kHz only between the workers from transport companies and the workers from the remaining categories, a fact that may be attributed to different ways of exposure to noise.
- The left ear presented worse audiometric thresholds than the right ear, for all evaluations, regardless of the professional category.
- Among the four groups, there was a significant worsening of arithmetical means at 3, 4, and 6 kHz due to exposure time, in all professional categories analyzed. However, this worsening presented a slow and progressive course, since—in comparisons between groups—it remained only significant between groups having less than five years and over 15 years of exposure to noise.
- In addition to noise exposure, other factors must be considered, such as the increase in the average age in groups that are more exposed to noise, along with other possible concurrent causes. To discuss these questions, further studies are necessary.

## Figures and Tables

**Table 1:** Descriptive analysis and comparison of the arithmetic means, in dB (HL), for the tonal thresholds at the audiometric frequencies of 3, 4, and 6 kHz for each ear, establishing a function and comparing the evaluated sides (profile test by contrasts) (*N* = 2.140).

Companies	Variable	*N*	Mean	SD	Minimum	Median	Maximum
Footwear *P* = 0.3619	R_Mean346 L_Mean346	266 266	10.78 11.21	8.05 8.73	−1.70 −3.30	10.00 10.00	53.30 50.00

Beverage *P* < 0.0001	R_ Mean346 L_ Mean346	150 150	11.60 13.69	9.63 10.57	−3.33 −1.67	10.00 10.84	70.00 63.33

Ceramics *P* < 0.0001	R_ Mean346 L_ Mean346	234 234	11.36 12.98	9.51 11.18	−3.30 −5.00	8.30 10.00	58.30 63.30

Metallurgical *P* < 0.0001	R_Mean346 L_Mean346	1254 1254	11.39 13.03	10.77 10.96	−3.33 −3.33	8.33 10.00	90.00 10.00

Transport *P* < 0.0001	R_Mean346 L_Mean346	236 236	15.66 17.03	10.67 11.44	0.00 −1.67	13.33 15.00	63.33 60.00

**Table 2:** The arithmetic means, in dB (HL), of the tonal thresholds at the frequencies of 3, 4, and 6 kHz by age range (*N* = 1.582).

Range	Variable	*N*	Mean	SD	Minimum	Median	Maximum
15–25	R_Mean346	451	6.98	6.49	−3.33	6.67	70.00
	L_Mean346	451	8.53	8.11	−5.00	6.70	110.00

26–34	R_Mean346	462	8.65	6.31	−3.33	8.30	41.67
	L_Mean346	462	10.58	7.96	−3.33	10.00	60.00

35–44	R_Mean346	405	15.05	10.61	0.00	11.70	63.33
	L_Mean346	405	16.15	10.59	0.00	13.33	66.67

45–79	R_Mean346	264	21.84	14.10	1.67	18.33	90.00
	L_Mean346	264	23.90	13.83	3.33	20.00	80.00

**Table 3:** Relationship of the arithmetic means, in dB (HL), of the tonal thresholds at the frequencies of 3, 4, and 6 kHz by exposure time to noise (*N* = 2.140).

Exposure time (month)	Variable	*N*	Mean	SD	Minimum	Median	Maximum
≤60	R_Mean346	652	7.88	7.16	−3.33	6.70	70.00
	L_Mean346	652	9.25	8.63	−5.00	8.30	110.00

61–120	R_Mean346	489	9.12	7.09	−3.33	8.30	50.00
	L_Mean346	489	10.58	7.92	−3.33	8.33	53.33

121–180	R_Mean346	361	11.41	7.80	−3.30	10.00	63.33
	L_Mean346	361	12.80	8.28	−1.70	11.67	63.30

>180	R_Mean346	638	18.05	13.13	−1.67	15.00	90.00
	L_Mean346	638	19.77	12.95	0.00	16.67	80.00

*P* < 0.0001 for the exposure range effect and side effect.

## References

[1] Bernardi A. P. Z., Fiorini A. C., Costa E. A. (2006). *Perda auditiva induzida pelo ruído. Série A: Normas e Manuais Técnicos*.

[2] Lopes C. A., Almeida A. C. D., Mello A. D. P., Otubo K. A., Lauris J. R. P., Santos C. C. (2009). Caracterização dos Limiares Audiológicos em Trabalhadores de Urnas Funerárias. *Arquivos Internacionais de Otorrinolaringologia*.

[3] Fechter L. D., Chen G.-D., Rao D. (2002). Chemical asphyxiants and noise. *Noise and Health*.

[4] Casali J. G. (1994). Seeking the sounds of silence. *Virginia Tech Research*.

[5] Teles R. D. M., Medeiros M. P. H. (2007). Perfil audiométrico de trabalhadores do distrito industrial de Maracanaú—CE. *Revista da Sociedade Brasileira de Fonoaudiologia*.

[6] Cordeiro R., Lima-Filho E. C., Nascimento L. C. R. (1994). Associação da perda auditiva induzida pelo ruído com o tempo acumulado de trabalho entre motoristas e cobradores. *Cadernos de Saúde Pública*.

[7] Russo I. C. P., Santos T. M. M., Busgaid B. B., Osterne F. J. (1995). Um estudo comparativo sobre os efeitos da exposição à música em músicos de trio elétrico. *Revista Brasileira de Otorrinolaringologia*.

[8] Araújo S. A. (2002). Perda auditiva induzida pelo ruído em trabalhadores de metalúrgica. *Revista Brasileira de Otorrinolaringologia*.

[9] Borger M. E., Branco A. B., Ottoni A. C. (2009). A influência do espectro de ruído na prevalência de Perda Auditiva Induzida por Ruído em trabalhadores. *Brazilian Journal of Otorhinolaryngology*.

[10] Miranda C. R., Dias C. R., Pena P. G. L. (1998). Surdez ocupacional em trabalhadores industriais da região metropolitana de Salvador, Bahia. *Revista Brasileira de Otorrinolaringologia*.

[11] Oishi N., Schacht J. (2011). Emerging treatments for noise-induced hearing loss. *Expert Opinion on Emerging Drugs*.

[12] Henderson D., Subramaniam M., Boettcher F. A. (1993). Individual susceptibility to noise-induced hearing loss: an old topic revisited. *Ear and Hearing*.

[13] Kryter K. D. (1983). Presbycusis, sociocusis and nosocusis. *Journal of the Acoustical Society of America*.

[14] Kasper K. C. F., Gómez M. V. S. G., Zaher V. L. (2005). O ruído como fator estressante na vida de trabalhadores dos setores de serralheria e marcenaria. *Arquivos Internacionais de Otorrinolaringologia*.

[15] Almeida S. I. C., Albernaz P. L. M., Zaia P. A., Xavier O. G., Karazawa E. H. I. (2000). História natural da perda auditiva ocupacional provocada por ruído. *Revista da Associação Médica Brasileira*.

[16] Caldart A. U., Adriano C. F., Terruel I. (2006). Prevalência da perda auditiva induzida pelo ruído em trabalhadores da indústria têxtil. *Arquivos Internacionais de Otorrinolaringologia*.

[17] Harger M. R. H. C., Barbosa-Branco A. (2004). Efeitos auditivos decorrentes da exposição ocupacional ao ruído em trabalhadores de marmorarias no Distrito Federal. *Revista da Associação Médica Brasileira*.

[18] Nudelmann A. A., Costa E. A., Seligman J., Ibanez R. N. (2001). *Perda auditiva pelo ruído*.

[19] Santos U. P. (1994). *Ruídos: Riscos e Prevenção*.

[20] Fernandes J. C. (2001). *A influência dos protetores auditivos na inteligibilidade da voz [Undergraduate Thesis]*.

[21] Skoet R., Olsen J., Mathiesen B., Iversen L., Johansen J. D., Agner T. (2004). A survey of occupational hand eczema in Denmark. *Contact Dermatitis*.

[22] Comitê Nacional de Ruído e Conservação Auditiva (1999). *Perda Auditiva Pelo Ruído Relacionada ao Trabalho*.

[23] Miedema H. M. E., Oudshoorn C. G. M. (2001). Annoyance from transportation noise: relationships with exposure metrics DNL and DENL and their confidence intervals. *Environmental Health Perspectives*.

[24] Muzet T. A. (2007). Environmental noise, sleep and health. *Sleep Medicine Reviews*.

[25] Van Kempen E., Babisch W. (2012). The quantitative relationship between road traffic noise and hypertension: a meta-analysis. *Journal of Hypertension*.

[26] Sørensen M., Andersen Z. J., Nordsborg R. B. (2012). Road traffic noise and incident myocardial infarction: a prospective cohort study. *PLoS ONE*.

[27] Stansfeld S. A., Matheson M. P. (2003). Noise pollution: non-auditory effects on health. *British Medical Bulletin*.

[28] Da Costa E. A., Castro J. C., Macedo M. E. G. (2008). Iris pigmentation and susceptibility to noise-induced hearing loss. *International Journal of Audiology*.

[29] Nageris B. I., Raveh E., Zilberberg M., Attias J. (2007). Asymmetry in noise-induced hearing loss: relevance of acoustic reflex and left or right handedness. *Otology and Neurotology*.

[30] Berg R. L., Pickett W., Linneman J. G., Wood D. J., Marlenga B. (2014). Asymmetry in noise-induced hearing loss: evaluation of two competing theories. *Noise and Health*.

